# NeuroSwift: computer vision-based system to assess the cognitive–motor speed of soccer players—preliminary findings

**DOI:** 10.3389/fspor.2025.1724873

**Published:** 2026-01-27

**Authors:** Fabián Moya-Vergara, Ignacio Barrera-Gutiérrez, Pablo Arriaza-Marholz, Eduardo Piñones-Zuleta, Teresa Valverde-Esteve, Juan García-Manso, Enrique Arriaza-Ardiles, Marcos Zúñiga-Barraza

**Affiliations:** 1 Doctoral Program in Physical Activity and Sport, Faculty of Physical Activity and Sport Sciences, University of Valencia, Valencia, Spain; 2 Physical Activity and Sports Research Laboratory, Universidad de Playa Ancha, Valparaíso, Chile; 3 Electronics Department, Universidad Técnica Federico Santa María, Valparaíso, Chile; 4 Department of Engineering Design, Universidad Técnica Federico Santa María, Valparaíso, Chile; 5 Department of Physical Education, Art and Music Teaching, University of Valencia, Valencia, Spain; 6 Department of Physical Education, University of Las Palmas de Gran Canaria, Las Palmas de Gran Canaria, Spain

**Keywords:** athletic performance, computer vision, decision-making, motor skills, psychomotor performance, reaction time, soccer, user–computer interface

## Abstract

**Background:**

Cognitive–motor speed (CMS) in soccer integrates perceptual–cognitive processing with motor execution, yet many tools lack this integration and have limited ecological validity. NeuroSwift was engineered as a computer vision-based automated analysis platform to standardize tactical stimuli and produce reproducible measurements.

**Methods:**

A 3 × 3 interaction surface, front-facing visual stimuli, and HD video were orchestrated by a web application. Twenty-nine players (15 professionals, 14 university athletes) completed 16 scenarios (8 offensive, 8 defensive). Visuomotor reaction speed (VMRS), displacement speed (DS), and response capacity (RC) were obtained, and cognitive–motor speed (CMS = VMRS + DS, in seconds) was computed. Normality and homogeneity were verified using Shapiro–Wilk and Levene’s tests. VMRS and DS were compared using independent-samples *t*-tests (Bonferroni *α* = 0.0167). RC and CMS were assessed using the Mann–Whitney *U* test. Effect sizes were estimated. All tests were two-tailed, and confidence intervals were estimated where applicable.

**Results:**

Professionals showed faster VMRS (0.77 ± 0.12 vs. 0.96 ± 0.12 s; *p* < 0.001; *d* = 0.79), whereas university players showed faster DS (0.64 ± 0.06 vs. 0.76 ± 0.11 s; *p* < 0.001; *d* = −0.71). RC favored professionals (median 100.00% vs. 93.75%; *Z* = 3.13; *p* < 0.001; *r* = 0.58). CMS tended to favor professionals (median 1.53 s vs. 1.61 s) without significance (*Z* = −0.544; *p* > 0.05; *r* = 0.10).

**Conclusion:**

NeuroSwift enabled standardized stimuli, automated footstep detection, and reproducible *in situ* laboratory metrics. Expertise was discriminated in perceptual–cognitive and decision components, supporting athlete monitoring, training prescription, and applied research.

## 1 Introduction

In collaboration–opposition sports, performance depends on the player's ability to perceive, process, decide, and execute actions according to the context, a skill referred to as cognitive–motor speed (CMS) (1–3). This ability distinguishes experts from average players (4) and has driven the development of methods and technologies aimed at its optimization (5–13).

However, two key limitations persist. First, methodological constraints: current tools lack contextualization and specific transfer, reducing their applicability in real game situations (14, 15). Second, technological barriers: advanced solutions such as *Footbonaut* (16, 17) or *SoccerBot360* (12) involve high costs and operational complexity, restricting their use to elite clubs (18). This combination creates a gap between performance science and everyday practice, limiting the assessment and training of perceptual–tactical and motor skills in real environments.

To bridge this gap, NeuroSwift has been developed (19) as a portable and low-cost technology that integrates computer vision and automated analysis (20) to measure and develop CMS through simulations of collaboration–opposition scenarios. Its design enables simultaneous assessment of reaction speed, cognitive processing, and motor execution, ensuring high ecological validity and democratizing access to advanced tools.

This report aimed to describe this solution and present preliminary results of its experimental application, evaluating its ability to differentiate performance between professional and amateur players. It was hypothesized that professionals would exhibit superior CMS indicators, reflected in shorter visuomotor reaction and movement times, as well as greater effectiveness in task execution.

## 2 Methods

### 2.1 Participants

A total of 29 male soccer players from different performance levels were included: 15 professional players from the Chilean First Division (age, 24.64 ± 6.21 years; body mass, 75.34 ± 7.58 kg; height, 177 ± 5 cm; weekly training volume, 11.87 ± 3.34 h) and 14 Chilean federated university players (age, 20.22 ± 1.77 years; body mass, 73.21 ± 8.00 kg; height, 178 ± 5 cm; weekly training volume, 6.86 ± 1.70 h). All participants were outfield players (defenders, midfielders, and forwards); goalkeepers were excluded due to the distinct perceptual–motor demands and specialized training associated with this position. Players who were not actively competing or who exhibited documented symptoms of post-concussion syndrome or uncorrected sensory deficits were also excluded.

Informed consent was obtained from all participants prior to the procedures, and no financial compensation was provided. The study was approved by the Ethics Committee of Universidad de Playa Ancha and conducted in accordance with the principles of the Declaration of Helsinki (1964), including the most recent recommendations adopted at the General Assembly in Fortaleza, Brazil (October 2013).

### 2.2 Instruments—NeuroSwift

**Figure 1:**
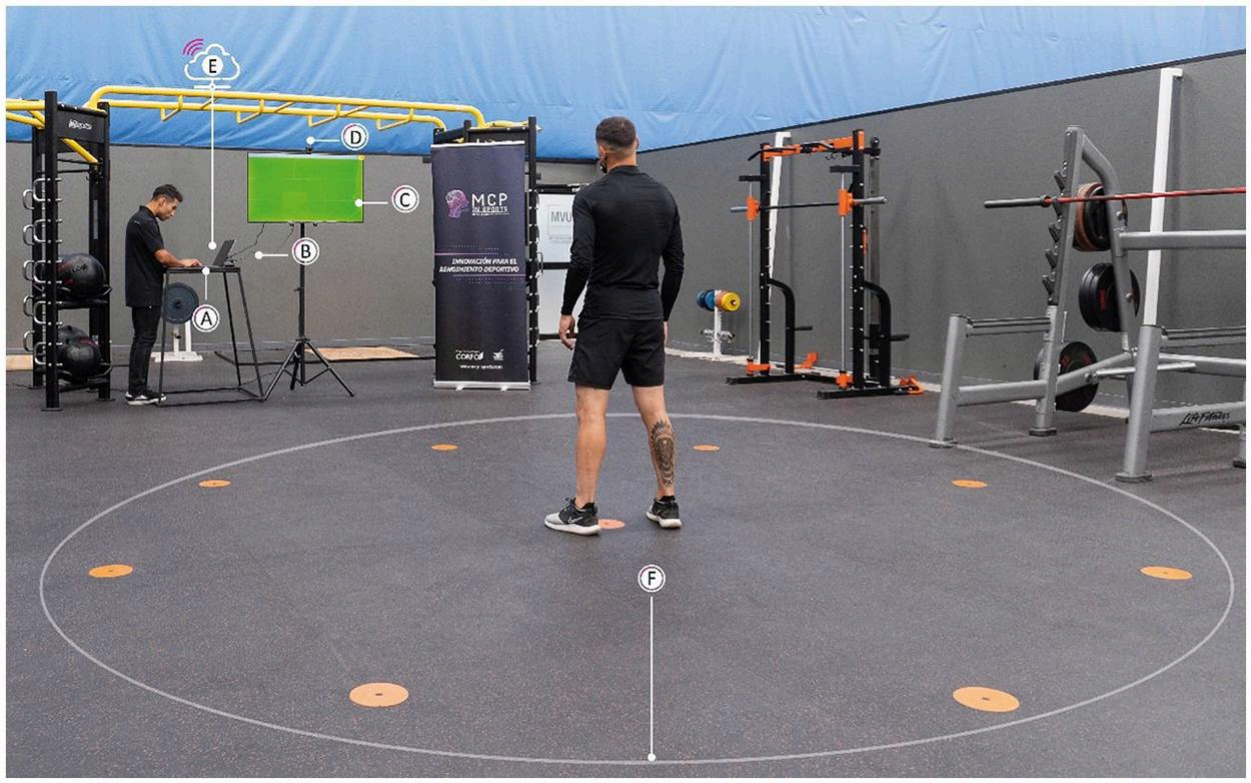
NeuroSwift technology tool. Schematic representation of the hardware configuration used to assess cognitive–motor speed in soccer players: **(A)** computer, **(B)** HDMI cable, **(C)** 50″ smart TV, **(D)** webcam, **(E)** Internet connection, and **(F)** 5 m × 5 m surface.

NeuroSwift is a computer vision-based technology designed to assess and train athletes' CMS. It integrates performance analysis software and a set of components that enable its operation. The software consists of a cloud-integrated web platform that allows the configuration, execution, and recording of an evaluative session and/or cognitive–motor training for soccer. The hardware of this system includes (A) computer, (B) HDMI connector cable, (C) smart TV (50″), (D) webcam, (E) Internet access, and (F) a clean, flat surface measuring 5 m × 5 m, on which nine circular markers (15 cm in diameter) are distributed and used as target points. Peripheral points are arranged at a radial distance of 2 m from the central point, with a central angle of 45° between two adjacent peripheral points. See Figure 1 for an illustration of the system hardware.

The software, operated by an evaluator from the computer, displays sequences of visual stimuli on the Smart TV screen that replicate game situations. The participant must perceive and interpret these stimuli, make the correct decision (attack or defend), and then move at high speed across the surface toward the designated target points.

**Figure 2:**
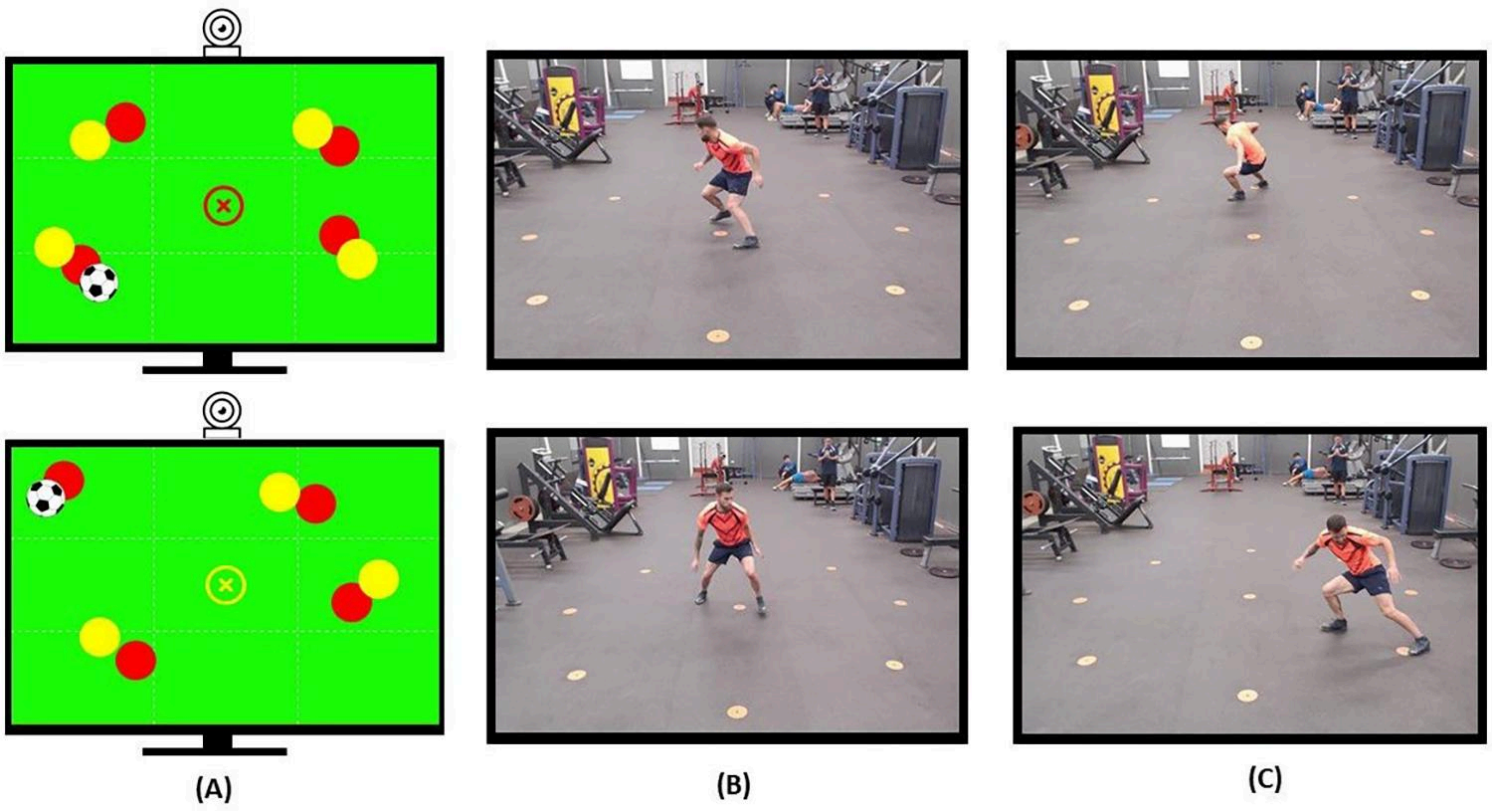
Example of visual stimuli and athlete responses. **(A)** Offensive scenario (top row) player moves backward to create a passing option; defensive scenario (bottom row) player moves diagonally to intercept an opponent. **(B)** Criterion for visuomotor reaction speed. **(C)** Criterion for displacement speed.

The motor–cognitive assessment (MCA) test consisted of a sequence of 16 game situations: 8 offensive (5 vs. 4) and 8 defensive (4 vs. 4). Offensive situations were based on the tactical principle of ball possession, requiring the participant to move into an unmarked free space to become a passing option for a teammate. Defensive situations were based on the tactical principle of individual marking, requiring the participant to move toward the opponent in possession of the ball to intervene in their action. Examples of offensive and defensive situations can be found in the top and bottom rows of Figure 2A. The total duration of the test was 1 min and 20 s.

During the test, data capture was performed using the webcam (synchronized with the software), positioned above the smart TV, which recorded all movements executed by the participant. This setup enabled the collection of various response times and accuracy/error percentages.

The experimental condition under which this measurement was conducted was classified as an *in situ* laboratory. According to McGuckian et al. (21), this condition allows participants free movement, employs non-living stimuli, and requires responses representative of the sport.

### 2.3 Variables

#### 2.3.1 Visuomotor reaction speed

Visuomotor reaction speed (VMRS) is defined as the ability of an individual to respond rapidly to unpredictable (visual) stimuli during competition. This variable represents the athlete's cognitive speed (22). Dependent variable: time (in seconds) required for the athlete to completely lift the attacking foot from the central circle of the platform. See the operational criterion in Figure 2B.

#### 2.3.2 Displacement speed

The ability to perform cyclic and acyclic movements without controlling an object (without the ball) at high speed. This variable represents the athlete's motor speed (22). Dependent variable: time (in seconds) required for the athlete to move toward the target point previously decided upon, after perceiving the game situation. The measurement considers the time elapsed from the moment the attacking foot leaves the central circle until the athlete steps on the target point. See the operational criterion in Figure 2C.

#### 2.3.3 Response capacity

Response capacity (RC) is defined as the athlete's ability to understand the game situation and produce an effective motor response (movement toward the target point required by the game situation). This variable represents decision-making ability. Dependent variable: percentage of correct responses, calculated as the number of correct actions divided by the total number of game situations and multiplied by 100.

### 2.4 Session execution and analysis workflow

All sessions were recorded at 30 fps and 1,920 × 1,080 resolution with a front-facing webcam mounted at approximately 2 m above the smart TV, covering the 3 × 3 target layout (Figure 1). Each session video is automatically synchronized to the stimuli log within NeuroSwift.

**Figure 3:**
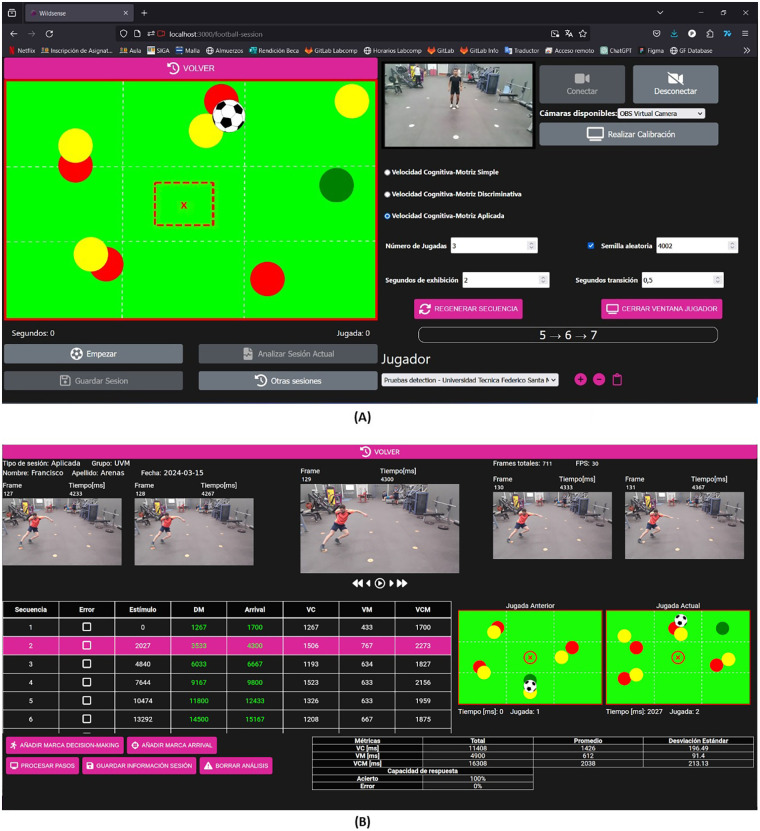
Main user interfaces of NeuroSwift. **(A)** Session recording interface presenting a stimulus contextualized to soccer; the correct answer is marked with a green circle, visible for the analyst, but hidden for the athlete. **(B)** Athlete performance analysis interface.

Calibration (action zone mapping): Before a session is executed, the session recording interface (Figure 3A) allows the evaluator to run a one-frame, semiautomatic calibration on a still image of the scene to locate the nine target zones. The procedure returns nine closed polygons (one per zone) in image coordinates and a planar homography from the image to the floor plane. The software first attempts automatic polygon detection via contrast-based region finding; if any zone is missing or misaligned, the evaluator clicks the six closest zone centers (near field). The software then computes the homography and projects the remaining three centers, re-centering each polygon onto its nearest high-contrast region. The evaluator can accept or nudge any polygon and confirm. This calibration has been routinely successful on indoor flooring, natural grass, and synthetic turf; reflective floors or highly non-uniform lighting may require the semiautomatic fallback.

Performance analysis: After calibration and session execution, the evaluator enters the athlete performance analysis interface (Figure 3B). Two workflows are available: (i) manual setting of movement onset (athlete reaction) and zone-arrival instants (first step on a target zone) or (ii) automatic video analysis, contingent on a valid calibration. The automatic analysis extracts BlazePose (23) foot landmarks (heel, ankle, foot index) per frame and derives left/right foot trajectories with light temporal smoothing. A step event is declared when complementary heuristics jointly indicate stable ground contact: (i) frame-to-frame displacement remains below a minimal-movement threshold (e.g., <2 px/frame) over a short stability window (e.g., ≥3 frames), (ii) a brief retrospective window confirms a change in displacement direction consistent with deceleration to rest, and (iii) the foot position remains within a compact bounding region across successive frames. To spatially ground the contact, a footprint polygon is formed by expanding from the foot keypoints within the BlazePose segmentation mask and then intersecting with the nine calibrated target zones; sufficient overlap (e.g., ≥20% area overlap) validates the zone of contact. For each stimulus–response cycle, NeuroSwift logs the movement onset from the central zone (reaction), the first validated contact in the target zone (arrival), and response correctness based on the intersected zone, yielding reaction time, arrival time, total cognitive–motor time, and accuracy for evaluator review.

For marker determination, the algorithm follows the same deterministic definitions exposed to the evaluator:

- Movement onset (visuomotor reaction start): first frame after stimulus presentation, with the athlete in contact with the central zone, in which the attacking foot ceases floor contact and is directed toward the target zone.
- Zone arrival: first frame in which either foot's segmentation polygon intersects the intended peripheral target-zone polygon (intersection computed in image space using calibrated target polygons).
- Response correctness: A trial is correct if the arrival zone equals the stimulus-defined zone; otherwise, it is incorrect. If no arrival occurs before the next stimulus, the trial is marked as no response.

Within the analysis interface, all automatically proposed time markers are displayed and can be accepted or edited frame accurately; edits immediately recompute the metrics. This algorithm version extends a previous work (20) by incorporating pose detection (BlazePose) and footprint segmentation for more precise zone intersection. The added cues improve robustness under partial self-occlusion, as full-body pose is tracked, and footprints are localized via segmentation seeded by foot keypoints. Preliminary tests indicate mean absolute errors of ∼4 frames (∼0.133 s) for reaction start and ∼1.6 frames (∼0.053 s) for zone arrival.

### 2.5 Throughput and quality control

On standard laptops (e.g., Intel Core i7 class, 16GB RAM), fully manual annotation of a session typically requires ∼20 min of frame-by-frame inspection. The assisted workflow completes the automated pass in ∼2–3 min, followed by a typical evaluator review of ∼2–3 min to correct residual imprecision. This represents a 70%–80% reduction in analyst time per session and enables practical analysis at scale. The analysis interface displays previous/current stimuli, proposed marks, and detected zone hits to streamline verification. All videos, timestamps, edits, and flags are stored on a central server with session-level metadata to support reproducibility.

### 2.6 Procedures

The MCA test was administered through the NeuroSwift platform in a single evaluation session dedicated exclusively to its application. Athletes were instructed on the required procedures and subsequently performed a standardized warm-up consisting of 4 min of continuous running at moderate intensity, followed by 4 min of joint mobility exercises, five 10 m sprints (two in a straight line and three with changes of direction), and 5 min of stretching. Each athlete completed the test three times (responding to a total of 48 game situations). The first two repetitions served as familiarization trials and were not included in the statistical analysis. To avoid learning effects or predictability, the system's randomization function was used, ensuring that each sequence comprised 16 different game situations (8 offensive and 8 defensive).

The locomotor demands of the test were limited to short-distance (2 m) multidirectional movements—frontal, lateral, and diagonal—requiring brief accelerations and decelerations rather than sustained sprints. A standardized rest interval of 6 min was established between repetitions, sufficient to restore performance after explosive movements of this magnitude (24). Additionally, players reported perceived exertion using a 1-to-10 scale, and subsequent repetitions were initiated only when an exertion value ≤2 was declared (25).

For execution, the athlete stood on the central circle of the target surface, 5 m from the smart TV, focusing attention on the screen where the evaluative task was displayed. The task began with a three-digit countdown (3–2–1), after which the game situations composing the sequence appeared. During the evaluative task, the TV dynamically displayed spheres representing players from both teams and a soccer ball associated with one of them, arranged to form a collaboration–opposition scenario. At the center of the image, a circle marked with the letter “x” represented the evaluated athlete. This figure was displayed in a specific color, either red or yellow. This initial phase lasted 1 s and served as the configuration time for the game situation and transition between plays. Subsequently, the game situation remained on screen for 2 s, constituting the visualization period during which the athlete was required to make a decision. The arrangement of players and the ball, the color of the figure representing the athlete, and the visualization time determined the action to be executed. To fulfill the test objective, the athlete observed the game situation, rapidly processed the perceived information, and then produced a motor response in the shortest possible time. This response consisted of moving toward one of the target points according to the contextual scenario. For an illustration, see Figure 2. Upon completion of the test, the evaluator stored the recorded information in the database for subsequent analysis.

### 2.7 Data analysis

Statistical analyses were performed using IBM SPSS, version 20.0 (Chicago, IL, USA). The sample was stratified into two groups: professional players and university players. Normality was assessed using the Shapiro–Wilk test, and homogeneity of variances was assessed using Levene's test.

Group differences were analyzed using Student's *t*-test for independent samples for the variables visuomotor reaction speed (VMRS) and displacement speed (DS) (assumptions met), whereas response capacity (RC) was analyzed using the non-parametric Mann–Whitney *U* test (assumptions not met).

To control the risk of Type I error across the three variables, Bonferroni correction was applied, adjusting the significance level to *α* = 0.0167 (0.05/3).

Additionally, an exploratory composite index termed CMS was calculated by summing VMRS and DS (both in seconds), aiming to provide an integrated measure of the time required for cognitive processing and motor execution. No weighting or normalization was applied, as both components share the same unit of measurement. This index (CMS) did not meet normality assumptions and was analyzed using the Mann–Whitney *U* test.

All tests were two-tailed. The significance level was set at *p* < 0.05 for general analyses and at *p* < 0.0167 for primary variables after Bonferroni correction. Effect size was interpreted using Cohen's *d* for parametric tests (thresholds: 0.20 small, 0.50 medium, 0.80 large, and ≥1.20 very large) and *r* = |Z|/√*N* for non-parametric tests (thresholds: 0.10 small, 0.30 medium, 0.50 large) (26).

## 3 Results

Comparisons between groups for VMRS and DS are presented in Table 1, while Table 2 shows RC and the exploratory composite index CMS. After Bonferroni correction (*α* = 0.0167), all primary variables remained significant (*p* < 0.001), confirming the robustness of the findings.

**Table 1:** Comparison of VMRS and DS between professional and university soccer players.

Variable	Professionals (mean ± SD) [CI]	University players (mean ± SD) [CI]	Mean difference	*p*-value	Adjusted *p*-value (Bonferroni’s)	Effect size (*d*)
VMRS (s)	0.77 ± 0.12 [0.709–0.831]	0.96 ± 0.12 [0.897–1.023]	−0.19	<0.001	<0.001	0.79
DS (s)	0.76 ± 0.11 [0.704–0.816]	0.64 ± 0.06 [0.609–0.671]	+0.12	<0.001	<0.001	−0.71

95% CI calculated for the mean using parametric estimation. Negative sign in *d* indicates direction of effect (greater times in professionals for DS). VMRS, visuomotor reaction speed; DS, displacement speed.

**Table 2:** Comparison of RC and CMS between professional and university soccer players.

Variable and ECI	Professionals (median; IQR) [CI]	University players (median; IQR) [CI]	Z	*p*-value	Effect size (*r*)
RC (%)	100.00; 6.25 [96.36–99.44]	93.75; 10.94 [85.54–93.46]	3.13	<0.001	0.58
CMS (s)	1.53; 0.08 [1.469–1.591]	1.61; 0.19 [1.521–1.679]	−0.544	>0.05	0.10

95% CI calculated for the median using non-parametric estimation. ECI, exploratory composite index; IQR, interquartile range; RC, response capacity; CMS, cognitive–motor speed.

### 3.1 VMRS

Professional players exhibited faster visuomotor reactions than university players (0.77 ± 0.12 vs. 0.96 ± 0.12 s), with a mean difference of −0.19 s (*p* < 0.001) and Cohen's *d* = 0.79. The 95% confidence intervals showed minimal overlap (0.709–0.831 vs. 0.897–1.023), supporting a clear separation between groups.

### 3.2 DS

Professionals recorded longer times than university players (0.76 ± 0.11 vs. 0.64 ± 0.06 s), with a mean difference of +0.12 s (*p* < 0.001) and Cohen's *d* = −0.71 (absolute magnitude ≈0.71). In this case, lower values indicate greater speed; therefore, university players were faster in this component.

### 3.3 RC

Professional players demonstrated a higher percentage of effective responses than university players (median = 100%, IQR = 6.25 vs. 93.75%, IQR = 10.94). The 95% confidence intervals did not overlap (professionals, 96.36–99.44; university players: 85.54–93.46), supporting group separation. The Mann–Whitney *U* test indicated a statistically significant difference (*Z* = 3.13; *p* < 0.001), with a large effect size (*r* = 0.58).

The composite index **CMS** (VMRS + DS) showed a trend toward shorter times in professionals (median = 1.53 s; IQR = 0.08; 95% CI 1.469–1.591 vs. 1.61 s; IQR = 0.19; 95% CI 1.521–1.679), but without statistical significance (Mann–Whitney *U*: *Z* = −0.544; *p* > 0.05; *r* = 0.10).

**Figure 4:**
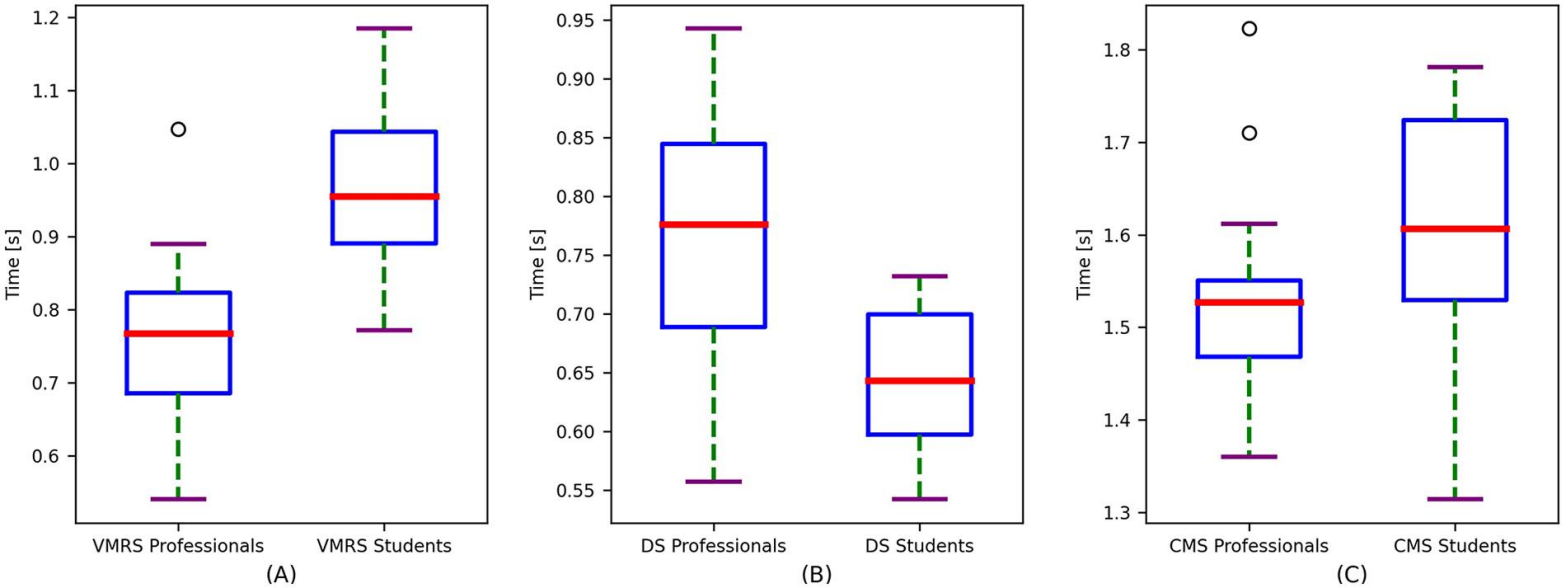
Distribution of **(A)** VMRS, **(B)** DS, and **(C)** CMS. Professionals exhibit lower medians in VMRS and CMS and higher medians in DS, consistent with the differences reported in the tables. The substantial overlap in CMS indicates no statistical significance. RC is omitted due to its distinct unit of measurement.

Figure 4 complements the inferential analysis by illustrating the distribution and dispersion of VMRS, DS, and the exploratory composite index CMS in each group.

## 4 Discussion

The present study described a tool (19) that enabled simultaneous analysis of cognitive and motor variables and, in turn, comparison of performance between soccer players of different competitive levels. Its application demonstrated that professional players exhibited clear advantages in VMRS and RC, whereas university players were faster in DS during task execution. Specifically, after Bonferroni adjustment (*α* = 0.0167), all three primary variables (VMRS, DS, and RC) remained significant (*p* < 0.001), with moderate-to-large effect sizes favoring professionals in VMRS and RC and favoring university players in DS. These results were obtained through an *in situ* laboratory task involving free movements and sport-representative responses (21), reinforcing ecological validity within a controlled design.

The cognitive advantages (VMRS and RC) displayed by higher-level players compared with their counterparts align with scientific evidence linking expertise to faster detection and processing of relevant information and more effective decision-making in dynamic contexts (2, 3, 27–34). Conversely, the superior performance of university players in DS may be explained by the nature of the task: very short (∼2 m), multidirectional displacements with changes of direction, where agility and explosiveness are more relevant (35) than speed contextualized within game phases. This pattern may favor younger players whose training emphasizes physical conditioning over tactical understanding. University players were 18% younger (4.42 years) than professionals, reflecting a substantial difference in developmental stage. Moreover, younger athletes may exhibit equal or superior performance in short explosive actions, indicating that physical advantage is not always exclusive to professionals (36).

Interestingly, the composite CMS index (VMRS + DS) did not reach significance between groups, suggesting a compensatory balance: the cognitive advantage (VMRS) of professionals is partially offset by the “pure” motor advantage (DS) of university players when both are aggregated into a single temporal indicator. This nuance is informative at an applied level: expertise gains appear to emerge more strongly in perceptual–cognitive and decision-making components than in very short-distance displacements per se, reinforcing the value of metrics representing different dimensions of performance rather than a single global measure for prescribing more specific and effective training (37).

Regarding the current debate on perceptual–cognitive skill training tools—whether extended reality and virtual reality (VR) (38–41), immersive video and VR (42), advanced visual training (5, 43), or neurofeedback (44)—all offer specific benefits but share common limitations: lack of standardization, absence of valid protocols, and scarce evidence of transfer to real competitive performance. Solutions proposed to simultaneously optimize cognition and technical efficiency, such as *Footbonaut* or *SoccerBot360* (12, 16, 17), correspond to high-cost systems with structural complexity yet still lack a tactical dimension. The findings of this study align with previous work (14, 41) questioning the transferability of generic brain-training devices and promoting task-representative designs for perceptual–cognitive evaluation and training (45, 47), while highlighting the need for further research (46). By combining tactical stimuli with automated measurement, NeuroSwift advances toward greater ecological validity compared with purely virtual or laboratory systems, while offering a cost-effective profile relative to high-hardware immersive platforms. This is achieved through the computer vision algorithm developed, which facilitates and reduces the cost of implementing this technology (20). In this sense, the study provides preliminary evidence of performance-level discrimination in an environment applicable for monitoring and prescription.

Methodologically, Bonferroni correction reduced the risk of Type I error without altering the significance of primary variables, reinforcing statistical robustness in a modest sample (*n* = 29). Operationally, the analysis pipeline (semiautomatic calibration, foot-strike event detection with BlazePose, and intersection with target zones) enabled the extraction of VMRS, DS, and RC with analyst review in a matter of minutes, reconciling frame-by-frame precision with practical feasibility for field use.

Overall, the results support that cognitive variables (VMRS and RC) are differentiating factors in high performance, whereas physical advantage may depend on task type, distance involved, and change-of-direction demands. From a practical perspective, this suggests dual prescriptions: (i) tasks that enhance perceptual–cognitive processing and decision-making under time pressure and (ii) highly specific reactive agility tasks to optimize perception–action transitions in reduced spaces.

## 5 Limitations

This pilot study presents several limitations. The single-session design and resource constraints prevented the estimation of test–retest reliability, inter-rater agreement, and comparative validation between manual and automated marking; algorithmic accuracy, although based on previous work, was not reassessed in this study. The small sample size (*n* = 29), appropriate for a *brief report*, limits generalizability and, together with homogeneous performance levels, may affect the interpretation of effect sizes. Differences in age and experience between groups may also have influenced the results. Although automatic randomization was applied, residual learning effects cannot be ruled out. Future research should expand the sample, incorporate reliability estimates, concurrent validation, and longitudinal studies linked to competitive performance.

## 6 Conclusion

The tool enabled discrimination of expertise levels in perceptual–cognitive components (VMRS and RC), whereas short-distance motor advantage (DS) emerged in university players, highlighting the need for disaggregated metrics. NeuroSwift complements evaluation and training with ecological validity and operational feasibility. Longitudinal monitoring, correlation with competitive performance indicators, and exploration in other sports (e.g., handball, hockey, basketball, rugby, tennis, and taekwondo) are recommended to expand its applicability beyond soccer.

## Data Availability

The raw data supporting the conclusions of this article will be made available by the authors, without undue reservation.
